# Migrant background and the impact of the COVID-19 pandemic on mental healthcare consultations among children and adolescents in Norway: a nationwide registry study

**DOI:** 10.1186/s12913-023-09666-3

**Published:** 2023-06-30

**Authors:** Ingeborg Hess Elgersma, Rannveig Kaldager Hart, Melanie Lindsay Straiton, Lars Johan Hauge, Anne Reneflot

**Affiliations:** 1 Centre for Epidemic Intervention Research, Norwegian Institute of Public Health, Oslo, Norway; 2 Department of Health and Inequality, Norwegian Institute of Public Health, Oslo, Norway; 3 Centre for Evaluation of Public Health Measures, Norwegian Institute of Public Health, Oslo, Norway; 4 Department of Mental Health and Suicide, Norwegian Institute of Public Health, Oslo, Norway

**Keywords:** Adolescence, Migrant background, COVID-19, Children, Healthcare use, Mental health

## Abstract

**Background:**

Despite concern about migrant children’s mental health and their access to mental healthcare services during the COVID-19 pandemic, this topic has attracted little research attention. This study aimed to examine the impact of the COVID-19 pandemic on the use primary and specialist healthcare services for mental health problems among children and adolescents with migrant background.

**Methods:**

Using event study models, we investigated the impact of lockdown and subsequent COVID-19 infection control measures on children’s health service use for mental health problems according to migrant background. Drawing on reimbursement data from Norwegian public healthcare providers we observe consultations in a pre-pandemic (2017–2019) and pandemic cohort (2019–2021) in primary and specialised care.

**Results:**

The pre-pandemic cohort included 77 324 migrants, 78 406 descendants of migrants and 746 917 non-migrants and the pandemic cohort included 76 830 migrants, 88 331 descendants and 732 609 non-migrants (age 6–19). The full cohorts were observed for mental healthcare use in primary care while a subsample (age 6–16) was observed for health care use in specialist care. Lockdown resulted in a dip in consultation volumes for mental disorders for all children, but this dip was relatively larger and more persistent for children with migrant background. After lockdown, consultation volumes rose more for non-migrant children than for children with migrant background. Consultations in primary healthcare peaked during January to April 2021 for non-migrants and descendants of migrants, but not for migrants (4%, 95% CI -4 to 11). In specialist care during the same period, consultations dropped by 11% for migrants (95% CI -21 to -1).

By October 2021, all mental health consultations in specialist care were up with 8% for non-migrants (95% CI 0 to 15), and down with -18% for migrants and -2% for descendants (95% CIs -31 to -5 and -14 to 10).

Migrant males experienced the largest reduction in consultations.

**Conclusions:**

Changes in consultation volumes among children with migrant background after lockdown were not as pronounced as for non-migrants, and at times actually decreased. This suggests that an increase in barriers to care emerged during the pandemic for children with a migrant background.

**Supplementary Information:**

The online version contains supplementary material available at 10.1186/s12913-023-09666-3.

## Background

The psychological burden on children and adolescents has been a returning concern during the COVID-19 pandemic [1, 2]. Non-pharmaceutical interventions to curb contagion reduced children and adolescents’ opportunities to socialize with friends, participate in leisure activities and to physically attend school. Additionally, economic recession and disruptions in mental healthcare services have been feared to impair children and adolescents’ mental health. In Norway, an eight-week lockdown was implemented on March 12, 2020. After a gradual reopening during the summer of 2020, social distancing restrictions fluctuated with the waves of contagion from the fall of 2020 through 2021. Recent studies focusing on the majority population in Norway have shown an increase in consultation frequency across a range of mental health outcomes [3, 4]. Studies relying on self-reported symptoms from the general population indicate that mental health deteriorates with prolonged lockdown [5–9].

Children and adolescents with migrant background may have been particularly vulnerable to the psychological burden of the COVID-19 pandemic: compared to the majority population, they were more likely to have an adult relative who was hospitalized with or died from COVID-19 [10] and to live in neighbourhoods with lengthy and wide-ranging non-pharmaceutical interventions. Migrants and their descendants are also overrepresented in crowded housing [11], making both social distancing and remote schooling more mentally straining [12]. Already before the pandemic, migrants faced greater barriers to accessing mental healthcare [13, 14]. Getting adequate healthcare when COVID-19 imposed restrictions are in force could be a larger challenge for migrants and their descendants than for the majority population. In a Norwegian cross-sectional survey taken 6 weeks after lockdown adult migrants were more likely to experience a reduction in follow-up from psychologists [15].

Despite concern for migrant children and adolescents’ welfare during the pandemic, few studies have examined the impact of the COVID-19 pandemic on their mental health [16]. The existing literature is further hampered by non-representative or low sample sizes, by focusing on the short- to medium-term phases of the pandemic, the lack of longitudinal data and of samples including children aged 12 years and younger. Two studies have compared changes in self-reported mental health between migrant and non-migrant adolescents in the first year of the pandemic [17, 18]. Ertanir and colleagues examined changes in mental health in a sample of 377 Swiss adolescents from the fall 2019 to the fall 2020 [17]. They found no noticeable differences between migrant and non-migrant adolescents. Similar results were reported in a study by Akkaya-Kalacy and colleagues based on a convenience sample of 853 Austrian native- and migrant-adolescents [18]. In a cross-sectional study of 3052 Austrian adolescents, Pieh and colleagues found higher levels of self-reported symptoms of depression and anxiety among migrant adolescents than non-migrants in February 2021 [19]. A related literature regards the differential impact by race in the US, describing that the mental health of children and adults from racial and ethnic minority groups have been disproportionately impacted by the pandemic [20, 21].

In this study we aim to examine the impact of the COVID-19 pandemic on the use of healthcare services for mental health problems among children and adolescents with migrant background. To better understand changes in healthcare use for mental disorders among children and adolescents with a migrant background, we compare these changes to the changes in healthcare use observed in non-migrant children and adolescents.

## Methods

### Approach

We use Norwegian population-wide data to examine changes in primary and specialist healthcare consultations for mental health symptoms and disorders in children aged 6–19 years old. We compare consultation volumes during the pandemic to pre-pandemic years, and we employ an approach that allows us to net out seasonal effects and period changes.

We utilize data from the Norwegian national emergency preparedness registry (BeredtC19). The register includes data from the Norwegian Control and Payment of Health Reimbursements Database (KUHR) and the National Patient Registry (NPR), as well as demographic data from Statistics Norway. Data on parents and children were linked through unique (de-identified) personal identifiers. The sample is restricted to all children aged 6–19 in 2018 or 2020.

### Health service use for mental health problems

In Norway, primary and specialist healthcare is free for all children aged 18 years and younger, lowering the threshold for seeking care also for low-income families. Data on primary healthcare use was retrieved from KUHR and specialist healthcare data from the NPR. KUHR includes consultation dates and diagnoses according to the International Classification of Primary Care (ICPC-2) with either a symptom or disorder code [22]. Primary healthcare encompasses in person, as well as digital, consultations with general practitioners (GPs) and emergency room visits. NPR includes consultation dates and diagnoses in accordance with the 10th edition of the International Classification of Diseases and Related Health Problems (ICD-10) [23].

Monthly dummy variables were constructed for every child in the sample, indicating as least one consultation for (i) all mental symptoms and disorders registered in primary care and specific diagnoses for ADHD, anxiety, depression, and sleep problems and (ii) all mental disorders in specialist care as well as specific diagnoses for ADHD, anxiety, depression and hospitalizations (see Table 2 for details on coding). Anxiety and depression were analysed together, due to the high level of comorbidity between these disorders [24, 25].

### Migrant background

Migrant background was constructed according to the definition from Statistics Norway by primarily relying on data from this source. When data on country of birth was missing for parents or the child from Statistics Norway we relied on data from the Norwegian population register. To be included in the sample, country of birth must be available for at least one parent as well as for the child. We distinguished between three categories:

1. Migrant children were defined as persons born abroad with two foreign-born parents.
2. Descendants of migrants were defined as children born in Norway with two foreign-born parents.
3. Persons born in Norway or born abroad, with one or two Norwegian-born parents were referred to as non-migrants.

Migrant background includes categories 1) and 2).

Low sample sizes (Table A1 in Supplementary materials) and accompanying low statistical power discouraged us from breaking down the migrant and descendant categories according to region or country of birth.

### Covariates

Age, sex and county of residence were extracted from the Norwegian population register and controlled for in all models when models were not run separately for these covariates.

### Statistical methods

To assert whether the COVID-19 pandemic impacted consultation volumes for children with migrant background differently than non-migrant children, we compare healthcare use between the three groups, before and during the pandemic. We construct a pre pandemic cohort, for whom we observe consultations from January 2017 to December 2019, age in defined by completed years is measured as of 1st January 2018, and inclusion in the sample is conditional on residing in Norway as of the same date. The pandemic cohort is residing in Norway as of 1st January 2020 and age in years is measured on the same date. For the pandemic cohort, consultations are observed from January 2019 to December 2021.

We first display trends in mental healthcare consultations for the pre-pandemic and the pandemic cohorts. We then fit multivariate event study models with controls for month and time in years to formally test, month by month, whether the change in use of healthcare services differs between the cohorts (see also Evensen and Hart et al. 2022 for a similar application):

$${y}_{i,t}={\sum }_{k=-14,k!=-1}^{21}{X}_{intervention}*1\left(t-t0=k\right){\beta }_{k}+{\sum }_{Y=-1}^{1}{\beta }_{Year}{X}_{Year,i,t}+{\sum }_{W=1}^{12}{\beta }_{Month}{X}_{month,i,t}+{\varvec{\beta}}{\varvec{X}}+\boldsymbol{ }\varepsilon ,$$

where y_i,t_ is 1 if person *i* has had at least one consultation in month *t,* otherwise 0*.* t0 is the first month of lockdown. The expression ${\sum }_{k=-14,k!=-1}^{21}{X}_{intervention}*1\left(t-t0=k\right){\beta }_{k}$ constructs dummy variables that take 1 if the observation is in the pandemic cohort, and *k* months away from March 2020, otherwise zero. The reference category is the month before lockdown, February 2020 (time t-1). All observations in the pre-pandemic cohorts fall into the reference category.

We control for calendar month to net out shared seasonal differences, and duration in years to handle shared secular change, by the following term ${\sum }_{Y=-1}^{1}{\beta }_{Year}{X}_{Year,i,t}$, which takes -1 if observation is in year 2017 (2019) in the pre-pandemic (pandemic) cohort, counting up to 1 for 2019 (2021). **X** is a vector of control variables, including region, age dummies, sex dummies, and a variable running from 0 to 1 indicating the proportion of Easter falling into that month.${\beta }_{k}$ shows how the trend in month k in the pandemic cohort differs from the trend in the same (relative) month in the pre-pandemic cohort. If consultation volumes in the pandemic cohort followed the same trend month-by-month, but at a different level than in the pre-pandemic cohort, estimates would be zero and non-significant.

These models are also used to assess whether the assumption of parallel trend before the interruption, alias the pandemic, holds.

Finally, we estimate difference-in-difference models, where we group months into six periods for the pandemic cohort (with measurements in the comparison sample always taken 24 months earlier): lockdown (March–May 2020), summer’20 (June–August 2020), fall’20 (September-December 2020), winter ‘21 (January-April 2021), summer’21 (June–August 2021) and fall’21 (September-December 2021). These models allows us to assess the effects of the pandemic.

While we rely on full population data, our statistical model splits data into several subgroups, reducing the statistical power of our model. Despite the caveats regarding reporting *p*-values in large samples [26], we therefore find it meaningful to report *p*-values in our application. However, acknowledging the limited information conveyed by *p*-values, we are careful to also report the magnitude of effects and their precision using confidence intervals (CIs), in accordance with the recommendation of Wassterstein et al. [27].

## Results

### Descriptive results

The pre-pandemic cohort included 77 324 migrants, 78 406 descendants and 746 917 non-migrants observed for mental healthcare use in primary care (age 6–18) and 56 445 migrants, 67 132 descendants and 582 148 non-migrants observed for mental healthcare use in specialist care (age 6–16). The pandemic cohort included 76 830 migrants, 88 331 descendants and 732 609 non-migrants observed for mental healthcare use in primary care (age 6–18) and 56 493 migrants, 75 708 descendants and 674 687 non-migrants observed for mental healthcare use in specialist care (age 6–16) (see table A1 in Appendix for details on age and sex).

The share with migrant background of the full sample, which includes children and adolescents aged 6–18, was 18% in the pre-pandemic cohort and 19% in the pandemic cohort. Migrants and descendants split roughly this category in two, with descendants making up the majority in both cohorts (Table 1).

**Table 1:** Descriptive statistics on consultations for mental health symptoms and disorders in primary and specialist healthcare and individual characteristics for children aged 6–19 years living in Norway

**A) Consultations in primary care**	**Jan 2017-Feb 2018**	**March 2018-Dec 2019**	**Jan 2019-Feb 2020**	**March 2020-Dec 2021**
Any mental symptom or disorder	0.91	1.11	0.97	1.27
	(0.66)	(0.81)	(0.68)	(0.84)
Anxiety/depression consultations	0.29	0.43	0.31	0.46
	(0.41)	(0.54)	(0.42)	(0.55)
Attention-deficit hyperactivity disorder consultations	0.07	0.09	0.08	0.1
	(0.11)	(0.13)	(0.11)	(0.12)
Sleep consultations	0.18	0.2	0.19	0.26
	(0.16)	(0.15)	(0.17)	(0.2)
Any consultation in primary care	11.41	11.45	11.31	11.01
	(4.16)	(4.66)	(3.99)	(4.17)
**B) Consultations in specialist care**				
Any mental disorder	1.69	1.99	1.72	2.02
	(0.86)	(1.06)	(0.87)	(1.22)
Anxiety/depression consultations	0.41	0.64	0.44	0.67
	(0.39)	(0.69)	(0.41)	(0.79)
Attention-deficit hyperactivity disorder consultations	0.64	0.70	0.64	0.75
	(0.45)	(0.47)	(0.45)	(0.49)
Hospitalizations	0.02	0.04	0.03	0.04
	(0.05)	(0.08)	(0.06)	(0.09)
**C) Sample characteristics**	**Primary care sample**		**Specialist care sample**	
	**2017–2019**	**2019–2021**	**2017–2019**	**2019–2021**
Age	12.51	12.52	10.97	11.03
	(3.73)	(3.68)	(2.64)	(2.62)
Female	0.49	0.49	0.49	0.49
	(0.5)	(0.5)	(0.5)	(0.5)
Non-migrants	0.82	0.81	0.82	0.81
	(0.38)	(0.39)	(0.39)	(0.39)
Migrants	0.09	0.09	0.08	0.08
	(0.29)	(0.28)	(0.28)	(0.27)
Descendants	0.09	0.10	0.10	0.11
	(0.29)	(0.30)	(0.30)	(0.31)
N (person months)	32 810 256	32 529 492	25 646 652	25 581 204
N (persons)	911 396	903 597	712 407	710 589

Panel A and B shows the percentage of children that had at least one contact of the given type in a given month with standard deviations in parentheses. For specialist care, age group 13–15 includes 16 year olds. Diagnoses are based on ICPC-2 codes, Chapter P for primary care, and ICD-10, Chapter F for specialist care (see Table 2)

**Table 2:** Codes and percent children with mental health problems and disorders according to the ICD-10 and ICPC-2 codes

		**Non-migrants**		**Migrants**		**Descendants**	
		2017–2019	2019–2021	2017–2019	2019–2021	2017–2019	2019–2021
**Primary care**	**ICPC-2 Code**						
Any mental symptom or disorder	All P	16.60	18.35	12.74	13.25	10.99	11.96
Anxiety/depression consultations	P74, P76, P79, P82, P01, P02, P03	6.68	7.40	5.74	5.94	3.61	4.11
Attention-deficit hyperactivity disorder	P81	1.97	2.25	2.23	1.93	1.38	1.46
Sleep consultations	P076	3.04	3.67	0.86	1.07	0.94	1.09
All consultations	All codes	88.86	88.60	83.56	82.77	85.99	86.17
**Specialist care**	**ICD-10 Code**						
Any mental disorder	All F	9.67	9.97	5.98	5.89	5.86	5.99
Anxiety/depression consultation	F32, F33, F40, F41, F43, F93.0, F93.1, F93.2	2.52	2.68	2.02	2.02	1.26	1.26
Attention-deficit hyperactivity disorder	F90	3.36	3.72	0.98	1.04	1.08	1.19
Hospitalizations	All F	0.70	0.72	0.56	0.54	0.40	0.40

The table gives the percentage of children that had at least one contact of the type in the given years

**Fig. 1:**
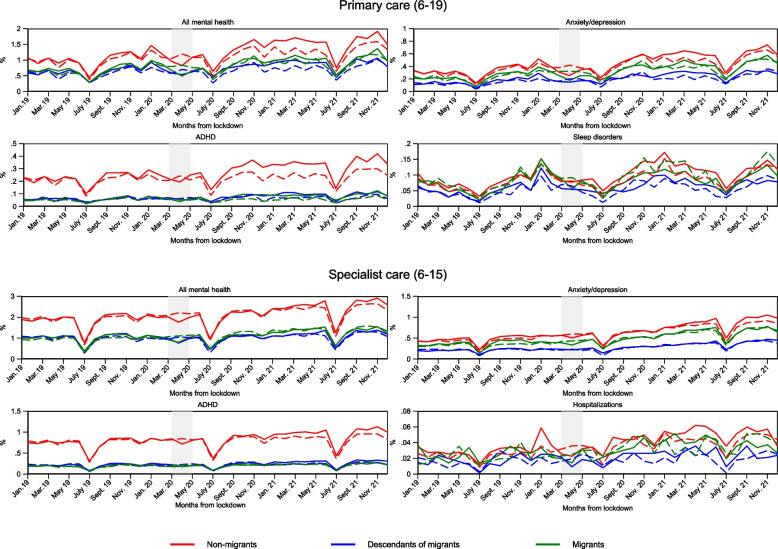
Consultations for mental health problems according to migrant background between January 2017(2019)-December 2019(2021) dashed (solid) lines. Percent of children with at least one consultation for mental health problems/disorders in primary and specialist healthcare in a given month. Diagnoses are based on ICPC-2 codes Chapter P for primary care, and ICD-10 Chapter F for specialist care (see Table 2). Separate calculations by migrant background and treatment group. The shaded area indicates the full lockdown period. The x-axis refers to the measurement time for the main sample (full lines). Dashed lines refer to the comparison groups, observed January 2017-December 2019. For the comparison sample, all measurements are made 24 months earlier

Figure 1 shows the monthly percentages with at least one consultation in primary and specialist healthcare for mental health symptoms and disorders from January 2019 to December 2021 in the pandemic cohort (full lines), compared to the similar percentages in the pre-pandemic cohort for January 2017 to December 2019 (dashed lines) by migrant background. The lockdown period in 2020 (March–April) is indicated by a shaded area.

Consultation volumes in primary and specialist care vary by season and with dips in school holidays (e.g., July and December). We observe an upward trend for all outcomes in both primary and specialist care. Before lockdown, trends are largely similar in the pandemic and pre-pandemic cohorts, fulfilling the assumption of parallel trends. During lockdown, consultations drop in the pandemic cohort, then quickly recuperate to the level of the pre-pandemic cohort and surpass the pre-pandemic level from January to May 2021 and September to December 2021.

Non-migrant children displayed the highest consultation volumes across both cohorts and across consultations in primary and specialist care. 16.6% of the pre pandemic cohort, and 18.4% of the pandemic cohort had a consultation in primary care for any mental symptom or disorder during the three years they were observed. The corresponding figures were 12.7% and 11.0% for the pre-pandemic migrant and descendant cohort and 13.3% and 12.0% for the pandemic migrant and descendant cohort (Table 2). The differences between non-migrant children and children with migrant background were particularly evident for ADHD diagnoses in primary and specialist care, and all mental healthcare in specialist care.

Migrants and descendants of migrants mostly exhibit similar consultation volumes, but for e.g. anxiety/depression in primary and specialist care as well as hospitalizations, descendants of migrants display lower volumes than migrant children and adolescents.

### Multivariate results

**Fig. 2:**
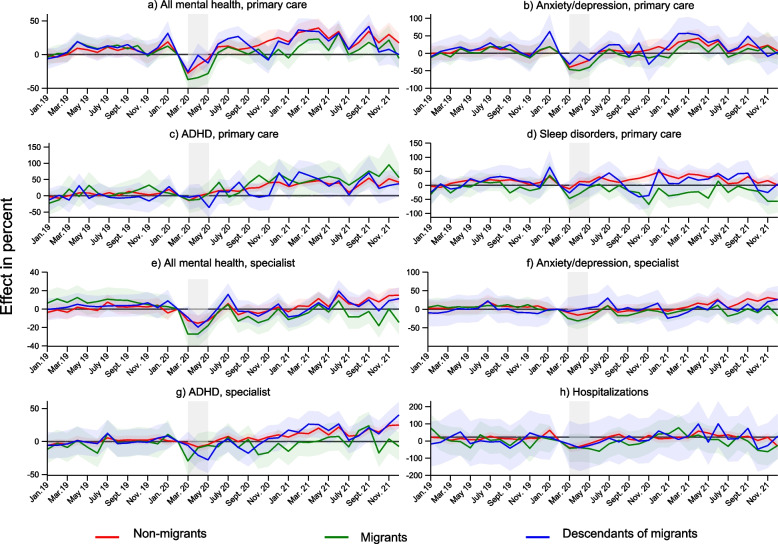
Changes in consultation volumes for mental health problems January 2019-December 2021 according to migrant background. Results from separate event study models by migrant background. Complete lines show coefficients, and shaded areas their 95% confidence intervals. Coefficients and confidence intervals are scaled to the pre-lockdown level in the main sample (see Table 1). The outcome is the monthly propensity of having at least one consultation of the type mentioned in the panel headers. Diagnoses are based on ICPC-2 codes Chapter P for primary care, and ICD-10 Chapter F for specialist care (see Table 2). The x-axis refers to the measurement time for the main sample. For the comparison sample, all measurements are taken 24 months earlier. Age group 13–15 includes 16-year-olds for specialist care. Models control for duration in years, sex, municipality, month and easter holidays

Figure 2 shows changes in the monthly probability of having one or more consultations in primary or specialist care. During the lockdown period, this probability decreased for all outcomes in primary and specialist care for all three groups of children, but the decrease was larger for migrants, except for ADHD in primary care. In figures, consultation volumes for all psychological disorders were down with 37% (95% CI -50 to -24) for migrants and 26% for descendants (95% CI -40 to -12) in primary care and 27% for migrants (95% CI -40 to -14) and 9% for descendants (95% CI -21 to 2) in specialist care.

In June and July 2020, following lockdown, we see a clear rebound effect regardless of migrant background for most outcomes in specialist care and, slightly more prominently, in primary care. By September 2020 consultation volumes have returned to pre-pandemic levels in primary and specialist care for non-migrants and descendants while migrants have lower consultation volumes in specialist care than the pre-pandemic trend (-14% in October 2020; 95% CI -28 to -2).

From December 2020, a new increase in consultation volumes is observed in both primary and specialist care, resulting in a peak for consultations in primary care in March 2021, coinciding with reinstated COVID-19 restrictions. Consultation volumes for all psychological disorders in primary care were up with 21% (95% CI 5 to 39) for migrants, 34% for descendants (95 CI 16 to 53) and 35% for non-migrants (95% CI 21 to 50).

By July 2021, consultation volumes were again returning to pre-pandemic levels across the board. Autumn 2021 saw an increase in consultations in primary care for all three groups. In specialist care, however, there were signs of divergence between migrants and partly descendants on the one side and non-migrants on the other. In October 2021, all mental health consultations in specialist care were up with 8% for non-migrants (95% CI 0 to 15), and down with -18% for migrants (95% CI -31 to -5) and -2% for descendants (95% CI -14 to 10).

Migrant consultation volumes for sleep disorders in primary care were deviating from these general trends during autumn 2020 and 2021 and remained mostly below pre-pandemic levels (Tables 3 and 4).

**Table 3:** Difference-in-difference estimates of change in the monthly probability of healthcare consultations in primary healthcare with 95% confidence intervals

		Any mental symptom or disorder				Anxiety/depression				ADHD				Sleep disorder				All primary consultations			
		Beta	CI Lower	CI Upper	p	Beta	CI Lower	CI Upper	p	Beta	CI Lower	CI Upper	p	Beta	CI Lower	CI Upper	p	Beta	CI Lower	CI Upper	p
Non-migrants	Lockdown	**-22.0**	-27.9	-16.0	< 0.001	**-33.1**	-43.8	-22.4	< 0.001	**-10.9**	-15.8	-5.9	< 0.001	**-9.3**	-16.9	-1.7	0.016	**-29.1**	-32.7	-25.5	0.000
	Summer '20	1.9	-4.1	7.9	0.539	-2.8	-13.8	8.2	0.613	**6.6**	1.4	11.9	0.013	3.2	-4.9	11.4	0.434	1.7	-1.9	5.3	0.351
	Fall '20	**7.7**	1.2	14.3	0.020	0.5	-10.9	11.9	0.928	**21.0**	15.1	26.9	< 0.001	**13.4**	6.1	20.7	< 0.001	**-6.4**	-9.4	-3.3	0.000
	Winter '21	**21.9**	15.1	28.6	< 0.001	**18.5**	6.5	30.5	0.002	**28.7**	23.1	34.3	< 0.001	**17.6**	10.1	25.0	< 0.001	**-9.2**	-12.3	-6.0	0.000
	Summer '21	**10.8**	4.7	17.0	0.001	5.7	-5.1	16.6	0.302	**19.0**	13.1	24.9	< 0.001	3.1	-4.1	10.2	0.400	-1.7	-4.8	1.5	0.297
	Fall '21	**14.8**	7.3	22.4	< 0.001	8.0	-5.5	21.6	0.245	**32.1**	25.7	38.5	< 0.001	0.0	-8.0	8.0	0.996	**11.4**	7.7	15.1	0.000
Migrants	Lockdown	**-35.6**	-42.7	-28.6	< 0.001	**-39.8**	-51.1	-28.6	< 0.001	-16.1	-37.7	5.5	0.144	**-18.0**	-34.3	-1.6	0.031	**-38.3**	-40.8	-35.7	0.000
	Summer '20	-3.2	-10.2	3.8	0.369	-7.6	-19.1	3.9	0.195	7.0	-14.9	28.8	0.532	-9.3	-23.9	5.2	0.209	**-4.9**	-7.4	-2.3	0.000
	Fall '20	-5.4	-13.0	2.2	0.167	-11.5	-24.0	1.1	0.073	**37.8**	13.8	61.9	0.002	**-20.0**	-35.9	-4.0	0.014	**-19.4**	-21.8	-17.0	< 0.001
	Winter '21	3.7	-4.0	11.3	0.348	8.2	-4.4	20.8	0.202	**37.7**	13.8	61.5	0.002	**-17.9**	-34.3	-1.4	0.033	**-18.0**	-20.7	-15.4	< 0.001
	Summer '21	0.4	-6.5	7.4	0.900	-2.5	-13.9	8.9	0.671	**36.4**	12.5	60.3	0.003	-11.9	-27.1	3.4	0.128	**-4.2**	-6.7	-1.7	0.001
	Fall '21	1.2	-7.4	9.9	0.778	3.1	-11.7	17.8	0.684	**55.6**	27.8	83.5	< 0.001	**-22.4**	-38.6	-6.2	0.007	-0.3	-3.1	2.4	0.818
Descendants	Lockdown	**-23.8**	-32.8	-14.9	< 0.001	**-34.0**	-53.3	-14.7	0.001	-16.6	-36.4	3.1	0.098	-19.3	-39.5	1.0	0.062	**-29.8**	-32.8	-26.9	< 0.001
	Summer '20	7.7	-1.2	16.7	0.090	-2.3	-21.3	16.8	0.816	16.9	-4.5	38.3	0.122	2.7	-17.0	22.3	0.788	-0.5	-3.2	2.3	0.750
	Fall '20	-4.6	-14.0	4.7	0.334	-19.4	-39.5	0.7	0.058	13.4	-8.0	34.8	0.220	**-22.6**	-41.8	-3.3	0.022	**-16.8**	-19.4	-14.3	< 0.001
	Winter '21	**17.1**	7.7	26.5	< 0.001	**24.6**	3.0	46.2	0.025	**44.2**	22.8	65.5	< 0.001	1.4	-19.1	22.0	0.891	**-16.4**	-19.2	-13.6	< 0.001
	Summer '21	8.3	-0.9	17.4	0.076	3.7	-16.4	23.9	0.716	**27.2**	6.9	47.6	0.009	7.9	-9.7	25.5	0.380	-0.8	-3.6	1.9	0.565
	Fall '21	1.8	-8.9	12.4	0.746	-0.8	-24.0	22.5	0.947	**35.6**	11.9	59.3	0.003	-11.5	-31.8	8.8	0.265	**7.4**	4.2	10.7	< 0.001

Results from difference-in-differences models, estimated separately by migrant background. Coefficients and confidence intervals are scaled to the pre-lockdown level in the main sample (see Table 1). The outcome is the monthly propensity of having at least one consultation of the given type. Diagnoses are based on IPCD codes Chapter P (see Table 2). For the comparison sample, all measurements are taken 24 months earlier. Models control for duration in years, sex, municipality, month, age category and easter holiday. Bold is added to emphasize statistically significant estimates (*p* < 0.05)

**Table 4:** Difference-in-difference estimates of change in the monthly probability of healthcare consultations in specialised healthcare with 95% confidence intervals

		Any mental disorder				Anxiety/depression				ADHD				Hospitalizations			
		Beta	CI Lower	CI Upper	p	Beta	CI Lower	CI Upper	p	Beta	CI Lower	CI Upper	p	Beta	CI Lower	CI Upper	p
Non-migrants	Lockdown	**-12.9**	-18.7	-7.0	< 0.001	**-15.5**	-28.1	-2.9	0.016	**-5.6**	-10.2	-1.1	0.016	**-42.7**	-57.4	-28.1	< 0.001
	Summer '20	-2.2	-8.1	3.6	0.454	-7.0	-19.8	5.9	0.289	1.9	-3.3	7.0	0.481	1.8	-13.1	16.7	0.811
	Fall '20	-2.3	-8.6	4.0	0.479	-6.9	-19.4	5.5	0.274	4.3	-0.7	9.3	0.092	-4.9	-21.3	11.4	0.552
	Winter '21	3.9	-3.8	11.7	0.321	0.0	-15.9	15.9	0.997	**12.0**	6.2	17.8	< 0.001	7.1	-13.5	27.7	0.498
	Summer '21	6.0	-2.5	14.4	0.166	6.4	-11.4	24.2	0.480	**10.6**	4.3	16.9	0.001	11.2	-9.5	31.9	0.289
	Fall '21	**11.1**	0.7	21.5	0.036	18.6	-3.2	40.4	0.095	**18.0**	10.3	25.6	< 0.001	-11.2	-31.2	8.8	0.271
Migrants	Lockdown	**-30.6**	-38.7	-22.5	< 0.001	**-30.2**	-44.1	-16.3	< 0.001	-13.8	-29.8	2.2	0.092	**-46.0**	-90.4	-1.7	0.042
	Summer '20	**-10.3**	-18.0	-2.7	0.008	-9.4	-22.4	3.6	0.158	-2.3	-17.8	13.1	0.769	-33.2	-79.6	13.2	0.161
	Fall '20	**-14.1**	-22.0	-6.1	0.001	-12.6	-25.7	0.4	0.058	-8.2	-24.0	7.6	0.308	2.4	-48.6	53.4	0.927
	Winter '21	**-10.8**	-20.6	-1.0	0.030	-7.8	-25.0	9.4	0.374	-1.3	-16.8	14.1	0.866	42.9	-10.1	95.8	0.113
	Summer '21	-10.1	-20.7	0.4	0.060	-10.3	-28.9	8.3	0.277	8.0	-7.8	23.7	0.322	0.5	-52.0	52.9	0.986
	Fall '21	**-14.9**	-27.7	-2.1	0.023	-9.1	-32.2	14.0	0.441	2.8	-14.0	19.7	0.740	-43.2	-97.3	11.0	0.118
Descendants	Lockdown	**-17.6**	-25.3	-9.9	< 0.001	-2.7	-21.2	15.8	0.778	**-18.7**	-30.4	-7.1	0.002	**-31.0**	-61.3	-0.6	0.045
	Summer '20	-0.5	-8.0	7.1	0.906	6.9	-12.4	26.1	0.484	-4.8	-17.1	7.5	0.448	-9.3	-37.0	18.4	0.509
	Fall '20	-6.5	-14.1	1.0	0.089	5.7	-12.5	23.8	0.541	-4.8	-16.7	7.1	0.427	5.7	-26.2	37.5	0.728
	Winter '21	-6.0	-15.0	2.9	0.187	-8.4	-32.2	15.4	0.489	**17.3**	5.1	29.6	0.006	18.6	-14.5	51.6	0.271
	Summer '21	2.6	-6.9	12.2	0.589	2.5	-23.8	28.7	0.855	11.0	-2.6	24.6	0.112	12.4	-19.1	43.8	0.441
	Fall '21	2.2	-8.1	12.5	0.670	13.5	-16.0	43.0	0.371	**20.9**	5.9	35.9	0.006	-4.4	-37.9	29.0	0.795

Results from Difference-in-differences models, estimated separately by migrant background. Coefficients and confidence intervals are scaled to the pre-lockdown level in the main sample (see Table 1). The outcome is the monthly propensity of having at least one consultation of the given type. Diagnoses are based on ICD-10 codes Chapter F (see Table 2). For the comparison sample, all measurements are taken 24 months earlier. Models control for duration in years, sex, municipality, month, age category and easter holiday. Bold is added to emphasize statistically significant estimates (*p* < 0.05)

The results from the difference-in-difference analysis confirm that children with migrant background experienced the largest relative dip in consultation volumes during lockdown, although confidence intervals overlap for most outcomes both in primary and specialised care. For any mental health problem in primary care the reduction for migrant children during lockdown was -36% (95% CI -43 to -29), while for non-migrants the reduction was -22% (95% CI -28 to -16) and the reduction for descendants was -24 (95% CI -33 to -15). The reduction was strongest for anxiety/depression. In specialist care the reductions in consultations for any mental disorder was -31% for migrants (95% CI -39 to -23), -13% for non-migrants (95% CI -19 to -7) and -18% for descendants (95% CI -25 to -9).

Winter 2021 consultation levels were significantly higher for mental health consultations in primary healthcare for non-migrants and descendants (22% and 17% respectively, 95% CI 15 to 29 and 8 to 27), while migrant children were on the pre-pandemic trend (4%, 95% CI -4 to 11). Similarly, fall 2021 saw an increase in consultation volumes for any mental symptom or disorder in primary care for non-migrant children of 15% (95% CI 7 to 22) while changes in consultations volumes for migrants and descendants were not statistically significant different from zero (*p*-values: 0.78 & 0.76). A noticeable exception was ADHD consultations, which saw significant increases for migrant children ranging between 36 and 55% in primary care from fall 2020 to fall 2021. A similar increase in ADHD consultations was seen for descendants of migrants.

### Subsample analysis

**Fig. 3:**
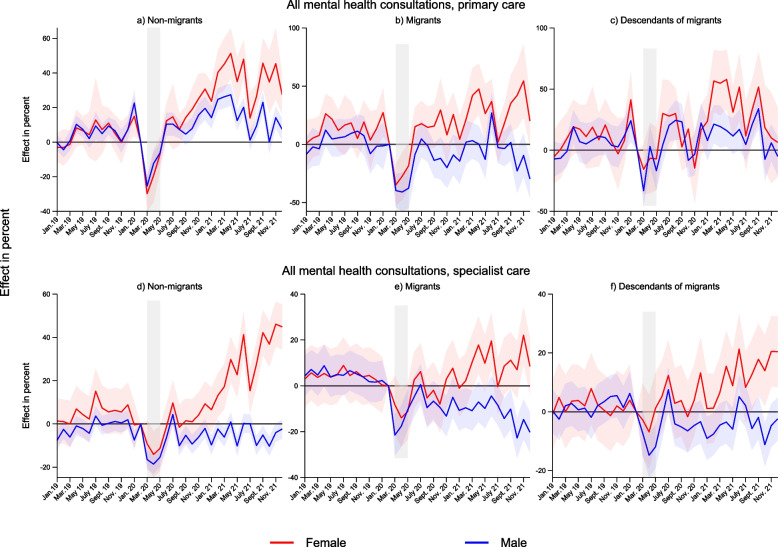
Changes in consultation volumes January 2019-December 2021 according to migrant background and sex. Results from separate event study models by sex and migrant background. Complete lines show coefficients, and shaded areas their 95% confidence intervals. Coefficients and confidence intervals are scaled to the pre-lockdown level in the main sample (see Table 1). The outcome is the monthly propensity of having at least one consultation of the type mentioned in the panel headers. Diagnoses are based on ICPC-2 codes Chapter P for primary care, and ICD-10 Chapter F for specialist care (see Table 2). The x-axis refers to the measurement time for the main sample. For the comparison sample, all measurements are taken 24 months earlier. Age group 13–15 includes 16-year-olds for specialist care. Models control for duration in years, sex, municipality, month and easter holidays

To explore in more detail the differences we observed by migrant background, we also ran these models separately by age and sex (Fig. 3 and Figure A1 and A2 in Supplementary materials). Females see considerable larger increases in consultation volumes than males after the pandemic hit both in primary and specialist care for all three groups of children. In specialised care, there is a clear statistically significant negative trend in consultation volumes after lockdown for migrant males. The decrease is substantial: At its lowest point, in October 2021, consultations for males in specialist care were down with 37% compared to pre-pandemic levels (95% CI -52 to -23).

**Fig. 4:**
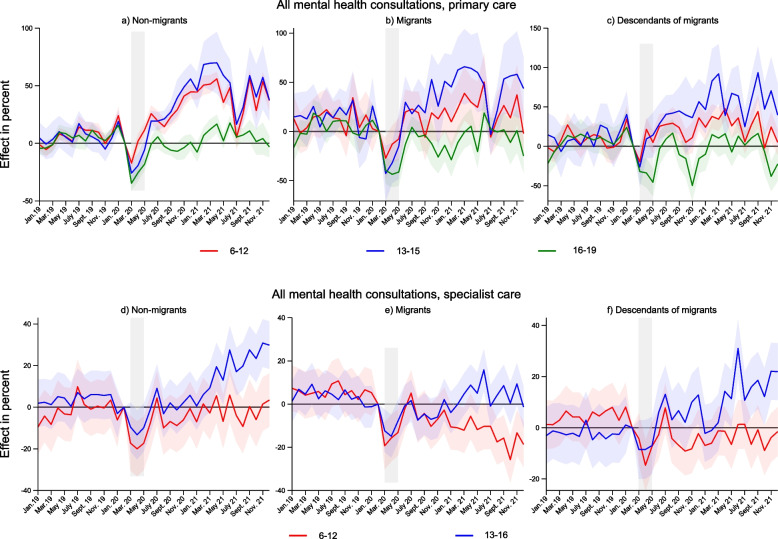
Changes in consultation volumes January 2019-December 2021 according to migrant background and age. Results from separate event study models by age and migrant background. Complete lines show coefficients, and shaded areas their 95% confidence intervals. Coefficients and confidence intervals are scaled to the pre-lockdown level in the main sample (see Table 1). The outcome is the monthly propensity of having at least one consultation of the type mentioned in the panel headers. Diagnoses are based on ICPC-2 codes Chapter P for primary care, and ICD-10 Chapter F for specialist care (see Table 2). The x-axis refers to the measurement time for the main sample. For the comparison sample, all measurements are taken 24 months earlier. Age group 13–15 includes 16-year-olds for specialist care. Models control for duration in years, sex, municipality, month and easter holidays

Figure 4 shows these trends according to age. Regardless of migrant background, 13–15-year-olds in primary care and 13–16-year-olds in specialist care see the largest increases in consultation volumes after lockdown. In the older age group, 16–19-year-old non-migrant adolescents mainly follow the pre-pandemic trend in primary healthcare, while we observe at times decreases in consultations for adolescents with migrant background in this age group. Furthermore, in specialist care migrant children aged 6–12 show a statistically significant drop in consultations from July to December 2021, reaching as low as a 26% reduction (95% CI -36 to -15) in October 2021.

No differences in trends by migrant background were seen according to whether the children and adolescents resided in the capital area (Oslo and Viken county) or in the rest of Norway (Figure A3).

## Discussion

### Principal findings

Using registry data covering three years of children’s healthcare service use for mental health problems, we were able to illuminate large differences according to migrant background and separate those from changes that arose after the pandemic. The descriptive trends revealed that both before and after the COVID-19 pandemic hit, children with migrant background used services for mental health problems less than children without migrant background.

We found that lockdown resulted in a significant and substantial reduction in the use of primary and specialist healthcare services for mental health problems, and that migrants faced the highest relative reduction (migrants: -36%, 95% CI -43 to -29; non-migrants: -22%, 95% CI -28 to -16; and descendants -24, 95% CI -33 to -15). After lockdown consultations returned to pre-pandemic levels within a couple of months.

In 2021, we observed two periods where consultations were significantly and substantially higher than the pre-pandemic trend: March to May and October to November. Migrant status modified this relationship: For most outcomes, fall 2021 showed a statistically significant and substantial increase in consultation volumes for children without migrant background (15% increase in mental health consultation in primary care, 95% CI 7 to 22). Meanwhile, changes to migrant children’s consultation volumes were often not statistically different from zero, or even negative. Consultation volumes for descendants of migrants more closely followed the trend for non-migrants, although also often not statistically different from zero either, except for a similar increase in ADHD consultations (a 44% increase in mental health consultations in primary care winter 2021, 95% CI 23 to 66).

Looking closer at which segments drove the differences observed by migrant background, we found that the non-significant, or negative, trend in consultation volumes in specialist care for migrants were mainly attributed to males and children aged 6–12 years.

### Interpretations and implications

On the whole, children with migrant background did not experience substantially higher consultation volumes in primary and specialist healthcare after lockdown than non-migrants. To the contrary, the trend was below pre-pandemic levels for migrant males and migrants aged 6–12 years for considerable periods in the fall 2020 and in 2021. This latter finding can be interpreted in at least two ways: On the one hand, there could be a real lack of change in demand for healthcare services for mental health problems for migrant youth after the pandemic, which suggests that the pandemic did not result in deteriorating mental health for this group. For migrants and their descendants, larger families and more dense social networks could have protected against poor mental health outcomes during social distancing. This interpretation contrasts with the finding of no clear differences by migrant background among Swiss adolescents [17], and slightly worse mental health outcomes for migrants and descendants as compared to the majority population among Austrian adolescents [18, 19]. These differences could be driven by differential burdens of social distancing, and/or different levels of discrimination across societies.

It is also possible, however, that our results differ from results based on self-reported health because they reflect increased barriers to access mental healthcare. Like in many other countries, the use of e-consultations increased steeply in the first phase of the pandemic, and some studies have indicated that vulnerable groups including migrants experience challenges in relation to e-consultation due to lack of digital and/or language skills and lack of private space [28, 29]. Furthermore, to the extent that children with migrant background seek mental healthcare by indication from the school system, such referrals may have become rarer during the pandemic, as e.g., contact with public health nurses and teacher became less frequent. In times of more remote schooling, lower health literacy among parents may translate into fewer referrals among migrants or descendants of migrants in particular. Related to this explanation, the heightened overall demand for specialist healthcare for severe and treatment-intensive diagnoses [4] could have meant that parental resources and health literacy had larger bearing on who gained access to specialist healthcare.

We found that differences in trends between children with migrant background versus children without such background were most discernible for migrant youth. Descendants of migrants are similar to non-migrants as they are both born and schooled in the Norwegian system and so language is less problematic than an adolescent who moved to Norway. However, much of help-seeking behaviour is likely learned from the home and perceptions of mental health may be very much influenced by their parents’ and others’ perceptions within their own community [30].

### Strength and weaknesses

A strength of our study is the full population data. Migrants and descendants have lower survey response rate and often reach a highly selected group in terms of language proficiency and socioeconomic status [31] and therefore cannot be analysed separately.

In the overall population, about 70% with a mental health diagnosis will have a primary care consultation related to mental health over a three year period [32]. As such, our data picks up a large, but not complete, share of the diagnosed. However, changes in consultation frequency could also have been due to changes in the capacity of the healthcare system. If the aim is to estimate the change in mental health during the pandemic, our study is limited in that we do not measure mental health directly. This is particularly important when comparing change during the pandemic among migrants, their descendants and others, as migrants already underutilize health services [13, 14], and the pandemic can have heightened the barriers to health service use more for migrants and descendants than the majority population.

As for the external validity of our results, some important features of the Norwegian context should be highlighted. First, the relatively low rates of COVID-19 death and severe morbidity, and moderate levels of contact reducing non-pharmaceutical interventions in place means that both the overall and any excess strain experienced by the migrant and descendant community, may be lower than in other western contexts. While increased disparities in unmet needs during the pandemic has been found in the US [20], a publicly financed and provided healthcare system may be better suited to cater to marginalized social groups than more insurance based systems, both in pandemic and ordinary periods.

## Conclusion

Contrary to our expectation, changes in consultation volumes for mental health problems among children with migrant background during the COVID-19 pandemic were not higher than for non-migrant children and adolescents. During some periods from March 2020 to December 2021, migrant children even faced reductions in consultation volumes compared to the pre-pandemic trend. These findings suggest an increase in barriers to care emerged during the pandemic for children with a migrant background. Our results suggest that national health authorities should address increasing disparities in access to mental health care in a health crisis such as the COVID-19 pandemic. Furthermore, the school and primary care should have a particular focus on children and adolescents with immigrant background and their mental health in the wake of the COVID-19 pandemic.

Finally, to get the full picture of how the mental health of migrant youth changed during the pandemic, and whether they receive adequate healthcare support, both data on self-reported health and use of health services is needed. Thus, it is crucial that this evidence is complemented with self-reported measures of mental health from nationally representative surveys that also cover migrant and descendant children and adolescents.

## Supplementary Information

**Additional file 1: Appendix A.** Supplementary results. **Table A1.** Sample characteristics and sample sizes in the pre-pandemic cohort and pandemic-cohort observed for ages 6-19 (primary care) and 6-16 (specialist care) according to migrant background. **Table A2.** Top 10 country background for migrants and descendants of migrants. Pre-pandemic cohort (age 6-19) in panel A) and pandemic-cohort (ages 6-19) in panel B). **Figure A1.** Results from separate event study models by migrant background (females). Complete lines show coefficients, and shaded areas their 95% confidence intervals. Coefficients and confidence intervals are scaled to the pre-lockdown level in the main sample (see Table 1). The outcome is the monthly propensity of having at least one consultation of the type mentioned in the panel headers. Diagnoses are based on ICPC-2 codes Chapter P for primary care, and ICD-10 Chapter F for specialist care (see Table A.1). The x-axis refers to the measurement time for the main sample. For the comparison sample, all measurements are taken 24 months earlier. Age group 13-15 includes 16-year-olds for specialist care. Models control for duration in years, sex, municipality, month and easter holidays. **Figure A2.** Results from separate event study models by migrant background (males). Complete lines show coefficients, and shaded areas their 95% confidence intervals. Coefficients and confidence intervals are scaled to the pre-lockdown level in the main sample (see Table 1). The outcome is the monthly propensity of having at least one consultation of the type mentioned in the panel headers. Diagnoses are based on ICPC-2 codes Chapter P for primary care, and ICD-10 Chapter F for specialist care (see Table A.1). The x-axis refers to the measurement time for the main sample. For the comparison sample, all measurements are taken 24 months earlier. Age group 13-15 includes 16-year-olds for specialist care. Models control for duration in years, sex, municipality, month and easter holidays. **Figure A3.** Results from separate event study models for capital area (Oslo and Viken countries) and the rest of Norway, by migrant background. Complete lines show coefficients, and shaded areas their 95% confidence intervals. Coefficients and confidence intervals are scaled to the pre-lockdown level in the main sample (see Table 1). The outcome is the monthly propensity of having at least one consultation of the type mentioned in the panel headers. Diagnoses are based on ICPC-2 codes Chapter P for primary care, and ICD-10 Chapter F for specialist care (see Table A.1). The x-axis refers to the measurement time for the main sample. For the comparison sample, all measurements are taken 24 months earlier. Age group 13-15 includes 16-year-olds for specialist care. Models control for duration in years, sex, municipality, month and easter holidays.

## Data Availability

The data that support the findings of this study are available from Norwegian Directorate of Public Health (Norwegian Patient Registry and Norwegian Control and Payment of Health Reimbursements Database), Statistics Norway (Fd-Trygd), and the Norwegian Tax Administration (National Population Register) but restriction apply to the availability of these data, which were used under license for the current study, and so are not publicly available. Data are however available from Anne Reneflot, anne.reneflot@fhi.no, upon reasonable request and with permission of Norwegian Directorate of Public Health, Statistics Norway and the Norwegian Tax Administration.

## References

[1] Golberstein E, Wen H, Miller BF (2020). Coronavirus disease 2019 (COVID-19) and mental health for children and adolescents. BMC Health Serv Res.

[2] Racine N, Cooke JE, Eirich R, Korczak DJ, McArthur B, Madigan S (2020). Child and adolescent mental illness during COVID-19: A rapid review. BMC Health Serv Res.

[3] Evensen M, Hart R, Godøy AA, Hauge LJ, Lund IO, Knudsen AKS (2022). Impact of the COVID-19 pandemic on mental healthcare consultations among children and adolescents in Norway: a nationwide registry study. BMC Health Serv Res.

[4] Surén P, Skirbekk AB, Torgersen L, Bang L, Godøy A, Hart RK (2021). Eating disorder diagnoses in children and adolescents in Norway before vs during the COVID-19 pandemic. BMC Health Serv Res.

[5] Mohler-Kuo M, Dzemaili S, Foster S, Werlen L, Walitza S (2021). Stress and mental health among children/adolescents, their parents, and young adults during the first COVID-19 lockdown in Switzerland. BMC Health Serv Res.

[6] Koenig J, Kohls E, Moessner M, Lustig S, Bauer S, Becker K (2021). The impact of COVID-19 related lockdown measures on self-reported psychopathology and health-related quality of life in German adolescents. BMC Health Serv Res.

[7] Li SH, Beames JR, Newby JM, Maston K, Christensen H, Werner-Seidler A (2021). The impact of COVID-19 on the lives and mental health of Australian adolescents. BMC Health Serv Res.

[8] Hafstad GS, Sætren SS, Wentzel-Larsen T, Augusti EM. Adolescents’ symptoms of anxiety and depression before and during the Covid-19 outbreak – A prospective population-based study of teenagers in Norway. Lancet Reg Health Eur. 2021;5:100093. 10.1016/j.lanepe.2021.100093. 10.1016/j.lanepe.2021.100093 PMC8454857 34557820

[9] Thorisdottir IE, Asgeirsdottir BB, Kristjansson AL, Valdimarsdottir HB, JonsdottirTolgyes EM, Sigfusson J (2021). Depressive symptoms, mental wellbeing, and substance use among adolescents before and during the COVID-19 pandemic in Iceland: a longitudinal, population-based study. Lancet Psychiatry.

[10] Indseth T, Grøsland M, Arnesen T, Skyrud K, Kløvstad H, Lamprini V (2021). COVID-19 among immigrants in Norway, notified infections, related hospitalizations and associated mortality: A register-based study. Scand J Public Health.

[11] Arnesen PK. Innvandrere bor trangere [Internet]. Ssb.no. 2020. Available from: https://www.ssb.no/bygg-bolig-og-eiendom/artikler-og-publikasjoner/innvandrere-bor-trangere. [cited 2022 Jul 13].

[12] Popyk A (2021). The impact of distance learning on the social practices of schoolchildren during the COVID-19 pandemic: reconstructing values of migrant children in Poland. Eur J Public Health.

[13] Straiton ML, Reneflot A, Diaz E (2014). Immigrants’ use of primary health care services for mental health problems. BMC Health Serv Res.

[14] Abebe DS, Lien L, Elstad JI (2017). Immigrants’ utilization of specialist mental healthcare according to age, country of origin, and migration history: a nation-wide register study in Norway. Soc Psychiatry Psychiatr Epidemiol.

[15] Mæland S, Bjørknes R, Lehmann S, Sandal GM, Hazell W, Rabben ÅK (2022). How the Norwegian population was affected by non-pharmaceutical interventions during the first six weeks of the COVID-19 lockdown. Scand J Public Health.

[16] Gibson B, Schneider J, Talamonti D, Forshaw M (2021). The impact of inequality on mental health outcomes during the COVID-19 pandemic: A systematic review. BMC Health Serv Res.

[17] Ertanir B, Kassis W, Garrote A (2021). Longitudinal Changes in Swiss Adolescent’s Mental Health Outcomes from before and during the COVID-19 Pandemic. Int J Environ Res Public Health.

[18] Akkaya-Kalayci T, Kothgassner OD, Wenzel T, Goreis A, Chen A, Ceri V (2020). The Impact of the COVID-19 Pandemic on Mental Health and Psychological Well-Being of Young People Living in Austria and Turkey: A Multicenter Study. Int J Environ Res Public Health.

[19] Pieh C, Dale R, Jesser A, Probst T, Plener PL, Humer E (2022). The Impact of Migration Status on Adolescents’ Mental Health during COVID-19. Healthcare.

[20] Thomeer MB, Moody MD, Yahirun J (2022). Racial and Ethnic Disparities in Mental Health and Mental Health Care During The COVID-19 Pandemic. J Racial Ethn Health Disparities.

[21] Xiao Y, Yip PSF, Pathak J, Mann JJ (2022). Association of Social Determinants of Health and Vaccinations With Child Mental Health During the COVID-19 Pandemic in the US. JAMA Psychiat.

[22] Global Family Doctor – WONCA Online [Internet]. Available from: https://www.globalfamilydoctor.com/groups/WorkingParties/wicc.aspx. [cited 2022 Jul 26].

[23] ICD-10 Version:2019 [Internet]. Available from: https://icd.who.int/browse10/2019/en. [cited 2022 Jul 26].

[24] Jacobi F, Höfler M, Strehle J, Mack S, Gerschler A, Scholl L (2015). Twelve-months prevalence of mental disorders in the German Health Interview and Examination Survey for Adults – Mental Health Module (DEGS1-MH): a methodological addendum and correction. Int J Methods Psychiatr Res.

[25] Plana-Ripoll O, Musliner KL, Dalsgaard S, Momen NC, Weye N, Christensen MK (2020). Nature and prevalence of combinations of mental disorders and their association with excess mortality in a population-based cohort study. World Psychiatry.

[26] Lin J, Lucas HC, Shmueli G (2013). Research commentary–too big to fail: large samples and the p-value problem. Inf Syst Res.

[27] Wasserstein RL, Lazar NA (2016). The ASA Statement on p-Values: Context, Process, and Purpose. Am Stat.

[28] Kaihlanen AM, Virtanen L, Buchert U, Safarov N, Valkonen P, Hietapakka L (2022). Towards digital health equity - a qualitative study of the challenges experienced by vulnerable groups in using digital health services in the COVID-19 era. BMC Health Serv Res.

[29] Cunningham NR, Ely SL, Barber Garcia BN, Bowden J (2021). Addressing Pediatric Mental Health Using Telehealth During Coronavirus Disease-2019 and Beyond: A Narrative Review. Acad Pediatr.

[30] Byrow Y, Pajak R, Specker P, Nickerson A (2020). Perceptions of mental health and perceived barriers to mental health help-seeking amongst refugees: A systematic review. Clin Psychol Rev.

[31] Ødegård G, Fladmoe A (2020). Are immigrant youth involved in voluntary organizations more likely than their non-immigrant peers to be engaged in politics? Survey evidence from Norway. Acta Sociologica.

[32] Torvik FA, Ystrom E, Gustavson K, Rosenström TH, Bramness JG, Gillespie N (2018). Diagnostic and genetic overlap of three common mental disorders in structured interviews and health registries. Acta Psychiatr Scand.

