# Supplementary material for: Migrant background and the impact of the COVID-19 pandemic on mental healthcare consultations among children and adolescents in Norway: a nationwide registry study
Source: BMC Health Serv Res. 2023 Jun 30;23:718. doi: 10.1186/s12913-023-09666-3 (PMC10314381; doi:10.1186/s12913-023-09666-3)
Supplement: Supplementary file 1 — Additional file 1: Appendix A. Supplementary results. Table A1. Sample characteristics and sample sizes in the pre-pandemic cohort and pandemic-cohort observed for ages 6-19 (primary care) and 6-16 (specialist care) according to migrant background. Table A2. Top 10 country background for migrants and descendants of migrants. Pre-pandemic cohort (age 6-19) in panel A) and pandemic-cohort (ages 6-19) in panel B). Figure A1. Results from separate event study models by migrant background (females). Complete lines show coefficients, and shaded areas their 95% confidence intervals. Coefficients and confidence intervals are scaled to the pre-lockdown level in the main sample (see Table 1). The outcome is the monthly propensity of having at least one consultation of the type mentioned in the panel headers. Diagnoses are based on ICPC-2 codes Chapter P for primary care, and ICD-10 Chapter F for specialist care (see Table A.1). The x-axis refers to the measurement time for the main sample. For the comparison sample, all measurements are taken 24 months earlier. Age group 13-15 includes 16-year-olds for specialist care. Models control for duration in years, sex, municipality, month and easter holidays. Figure A2. Results from separate event study models by migrant background (males). Complete lines show coefficients, and shaded areas their 95% confidence intervals. Coefficients and confidence intervals are scaled to the pre-lockdown level in the main sample (see Table 1). The outcome is the monthly propensity of having at least one consultation of the type mentioned in the panel headers. Diagnoses are based on ICPC-2 codes Chapter P for primary care, and ICD-10 Chapter F for specialist care (see Table A.1). The x-axis refers to the measurement time for the main sample. For the comparison sample, all measurements are taken 24 months earlier. Age group 13-15 includes 16-year-olds for specialist care. Models control for duration in years, sex, municipality, month and easter holid [file 12913_2023_9666_MOESM1_ESM.docx]

Supplementary material for “Migrant background and the impact of the COVID-19 pandemic on mental healthcare consultations among children and adolescents in Norway: a nationwide registry study”

[Appendix A: Supplementary results 2](#_Toc133611146)

# Appendix A: Supplementary results

**Table A1:** Sample characteristics and sample sizes in the pre-pandemic cohort and pandemic-cohort observed for ages 6-19 (primary care) and 6-16 (specialist care) according to migrant background

|  | | **Non-Migrants** | | **Migrants** | | **Descendants** | |
| --- | --- | --- | --- | --- | --- | --- | --- |
|  |  | 2017-2019 | 2019-2021 | 2017-2019 | 2019-2021 | 2017-2019 | 2019-2021 |
| Primary care (ages 6-19) | N (person) | 746 917 | 732 609 | 77 324 | 76 830 | 78 406 | 88 331 |
|  | Female N, % | 363 858, 0.49 | 357 063, 0.49 | 36 685, 0.47 | 37 236, 0.48 | 38 238, 0.49 | 43 113, 0.49 |
|  | Age mean, sd | 12.55, 4.03 | 12.57, 3.99 | 13.27, 3.91 | 13.36, 3.82 | 11.29, 4.01 | 11.28, 3.95 |
| Specialist care (ages 6-16) | N (person) | 582 148 | 574 687 | 56 445 | 56 493 | 67 132 | 75 708 |
|  | Female N, % | 283 551, 0.49 | 279 762, 0.49 | 27 572, 0.49 | 27 651, 0.49 | 32 770, 0.49 | 36 982, 0.49 |
|  | Age mean, sd | 11.01, 3.14 | 11.08, 3.13 | 11.51, 3.04 | 11.69, 3 | 10.18, 3.16 | 10.17, 3.08 |

**Table A2:** Top 10 country background for migrants and descendants of migrants. Pre-pandemic cohort (age 6-19) in panel A) and pandemic-cohort (ages 6-19) in panel B).

| **A) Cohort 2017-2019** | | | | | |
| --- | --- | --- | --- | --- | --- |
| **Migrant country backgrounds** | **N** | **Prop.** | **Descendant country backgrounds** | **N** | **Prop** |
| Poland | 9 011 | 0·10 | Somalia | 7 872 | 0·09 |
| Syria | 8 937 | 0·10 | Iraq | 6 996 | 0·08 |
| Somalia | 5 581 | 0·06 | Pakistan | 6 219 | 0·07 |
| Lithuania | 4 261 | 0·05 | Poland | 4 686 | 0·05 |
| Eritrea | 3 958 | 0·04 | Vietnam | 4 189 | 0·05 |
| Afghanistan | 3 846 | 0·04 | Sri Lanka | 3 579 | 0·04 |
| Thailand | 2 506 | 0·03 | Kosovo | 3 390 | 0·04 |
| Germany | 2 118 | 0·02 | Turkey | 3 237 | 0·04 |
| Iraq | 1 955 | 0·02 | Bosnia-Hercegovina | 2 396 | 0·03 |
| Philippines | 1 877 | 0·02 | Iran | 2 332 | 0·03 |
| **B) Cohort 2019-2021** | | | | | |
| **Migrant country backgrounds** | **N** | **Prop.** | **Descendant country backgrounds** | **N** | **Prop** |
| Syria | 10 396 | 0·12 | Somalia | 8 796 | 0·10 |
| Poland | 8 343 | 0·09 | Iraq | 7 617 | 0·09 |
| Somalia | 4 528 | 0·05 | Poland | 6 465 | 0·07 |
| Lithuania | 4 002 | 0·05 | Pakistan | 5 978 | 0·07 |
| Eritrea | 3 816 | 0·04 | Vietnam | 3 969 | 0·04 |
| Afghanistan | 2 356 | 0·03 | Kosovo | 3 464 | 0·04 |
| Thailand | 2 298 | 0·03 | Turkey | 3 129 | 0·04 |
| Philippines | 1 984 | 0·02 | Sri Lanka | 3 121 | 0·04 |
| Germany | 1 901 | 0·02 | Lithuania | 2 779 | 0·03 |
| Iraq | 1 666 | 0·02 | Afghanistan | 2 757 | 0·03 |

**
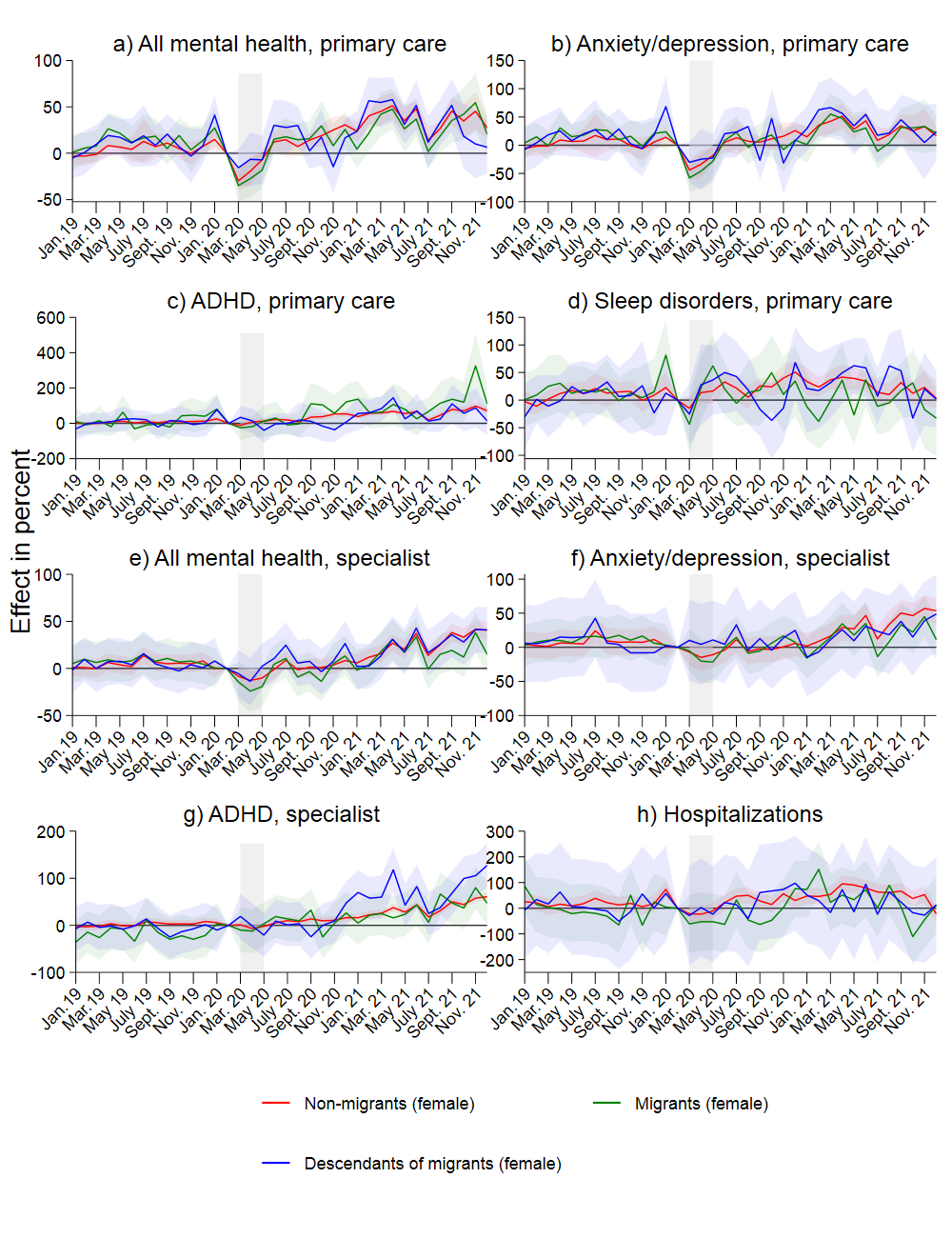
Figure A1:** Results from separate event study models by migrant background (females). Complete lines show coefficients, and shaded areas their 95% confidence intervals. Coefficients and confidence intervals are scaled to the pre-lockdown level in the main sample (see Table 1). The outcome is the monthly propensity of having at least one consultation of the type mentioned in the panel headers. Diagnoses are based on ICPC-2 codes Chapter P for primary care, and ICD-10 Chapter F for specialist care (see Table A.1). The x-axis refers to the measurement time for the main sample. For the comparison sample, all measurements are taken 24 months earlier. Age group 13-15 includes 16-year-olds for specialist care. Models control for duration in years, sex, municipality, month and easter holidays**.**

**
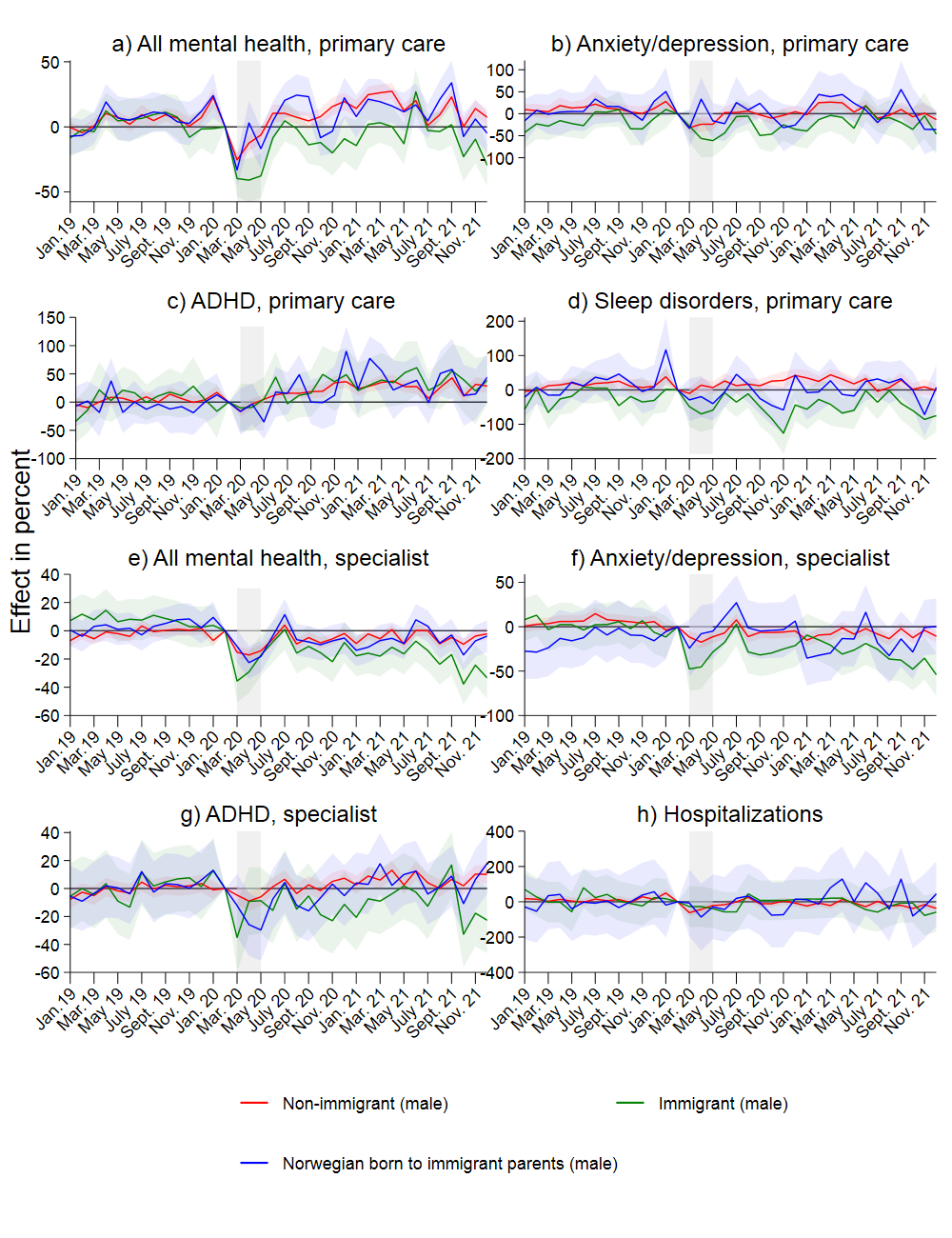
Figure A2:** Results from separate event study models by migrant background (males). Complete lines show coefficients, and shaded areas their 95% confidence intervals. Coefficients and confidence intervals are scaled to the pre-lockdown level in the main sample (see Table 1). The outcome is the monthly propensity of having at least one consultation of the type mentioned in the panel headers. Diagnoses are based on ICPC-2 codes Chapter P for primary care, and. ICD-10 Chapter F for specialist care (see Table A.1). The x-axis refers to the measurement time for the main sample. For the comparison sample, all measurements are taken 24 months earlier. Age group 13-15 includes 16-year-olds for specialist care. Models control for duration in years, sex, municipality, month and easter holidays**.**

**
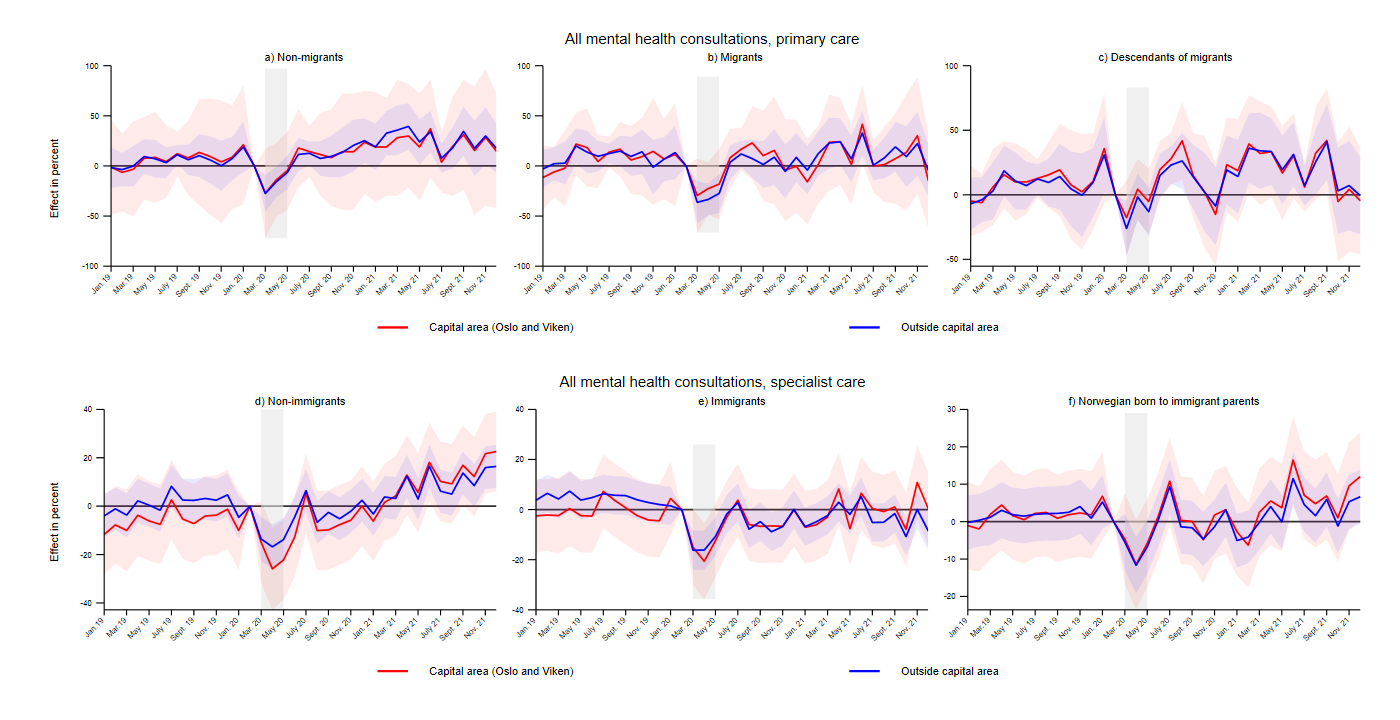
Figure A3:** Results from separate event study models for capital area (Oslo and Viken countries) and the rest of Norway, by migrant background. Complete lines show coefficients, and shaded areas their 95% confidence intervals. Coefficients and confidence intervals are scaled to the pre-lockdown level in the main sample (see Table 1). The outcome is the monthly propensity of having at least one consultation of the type mentioned in the panel headers. Diagnoses are based on ICPC-2 codes Chapter P for primary care, and. ICD-10 Chapter F for specialist care (see Table A.1). The x-axis refers to the measurement time for the main sample. For the comparison sample, all measurements are taken 24 months earlier. Age group 13-15 includes 16-year-olds for specialist care. Models control for duration in years, sex, municipality, month and easter holidays
